# Dietary Alpha-Lipoic Acid Alters Piglet Neurodevelopment

**DOI:** 10.3389/fped.2016.00044

**Published:** 2016-05-06

**Authors:** Austin T. Mudd, Rosaline V. Waworuntu, Brian M. Berg, Ryan N. Dilger

**Affiliations:** ^1^Piglet Nutrition and Cognition Laboratory, Department of Animal Sciences, University of Illinois, Urbana, IL, USA; ^2^Neuroscience Program, University of Illinois, Urbana, IL, USA; ^3^Mead Johnson Pediatric Nutrition Institute, Evansville, IN, USA; ^4^Division of Nutritional Sciences, University of Illinois, Urbana, IL, USA; ^5^Department of Animal Sciences, University of Illinois, Urbana, IL, USA

**Keywords:** alpha-lipoic acid, antioxidant, internal capsule, neurodevelopment, nutrition, piglet, neonatal

## Abstract

**Introduction:**

Alpha-lipoic acid (a-LA) is an antioxidant shown to ameliorate age-associated impairments of brain and cardiovascular function. Human milk is known to have high antioxidant capacity; however, the role of antioxidants in the developing brain is largely uncharacterized. This exploratory study aimed to examine the dose–response effects of a-LA on piglet growth and neurodevelopment.

**Methods:**

Beginning at 2 days of age, 31 male pigs received 1 of 3 diets: control (CONT) (0 mg a-LA/100 g), low a-LA (LOW) (120 mg a-LA/100 g), or high a-LA (HIGH) (240 mg a-LA/100 g). From 14 to 28 days of age, pigs were subjected to spatial T-maze assessment, and macrostructural and microstructural neuroimaging procedures were performed at 31 days of age.

**Results:**

No differences due to diet were observed for bodyweight gain or intestinal weight and length. Spatial T-maze assessment did not reveal learning differences due to diet in proportion of correct choices or latency to choice measures. Diffusion tensor imaging revealed decreased (*P* = 0.01) fractional anisotropy (FA) in the internal capsule of HIGH-fed pigs compared with both the CONT (*P* < 0.01)- and LOW (*P* = 0.03)-fed pigs, which were not different from one another. Analysis of axial diffusivity (AD) within the internal capsule revealed a main effect of diet (*P* < 0.01) in which HIGH-fed piglets exhibited smaller (*P* < 0.01) rates of diffusion compared with CONT piglets, but HIGH-fed piglets were not different (*P* = 0.12) than LOW-fed piglets. Tract-based spatial statistics, a comparison of FA values along white matter tracts, revealed 1,650 voxels where CONT piglets exhibited higher (*P* < 0.05) values compared with HIGH-fed piglets.

**Conclusion:**

The lack of differences in intestinal and bodyweight measures among piglets indicate a-LA supplementation does not impact overall growth, regardless of concentration. Additionally, no observed differences between CONT- and LOW-fed piglets in behavior and neuroimaging measures indicate a low concentration of a-LA does not affect normal brain development. Supplementation of a-LA at a high concentration appeared to alter white matter maturation in the internal capsule, which may indicate delayed neurodevelopment in these piglets.

## Introduction

Alpha-lipoic acid (a-LA), also known as thioctic acid, is an endogenously derived antioxidant found in animal and human cells alike, where it is a cofactor in mitochondrial dehydrogenase complexes. Although production of this dithiol occurs within the body, a-LA is known to be readily absorbed from the diet and is active in both aqueous and lipid-rich tissue. Lipoic acid is referred to as the “universal antioxidant” due to its multitude of functions including metal chelation, free radical quenching, antioxidant regeneration, and regulation of gene and protein expression (1). Moreover, provision of this molecule has been used for therapeutic treatments due its positive effects on diabetic polyneuropathy and age-associated impairments of cardiac and brain function (2, 3).

To date, much of research on a-LA supplementation has focused on its impact in the aging brain. Iron deposition that occurs in disease states such as Parkinson’s and Alzheimer’s increases occurrence of free radicals and peroxidation of polyunsaturated fatty acids (2). Dihydrolipoic acid (DHLA), the reduced form of a-LA, functions as a chelator of transition metals and may modulate the effects of excess iron on free radical production (4). Research shows supplementation of DHLA exerts a positive impact on the pathobiology of Alzheimer’s disease (5). Additionally, supplementation of a-LA attenuates age-related cognitive decline in aged mice, suggesting that age-related oxidative stress might be a contributing factor in impaired memory formation (6, 7). However, the largest randomized clinical trial to date using a combination of antioxidants, including a-LA, did not impact cerebrospinal fluid (CSF) biomarkers of tau and amyloid pathology and suggests treatment with this antioxidant combination results in faster cognitive decline. This clinical trial revealed differences in CSF F2-isoprostane levels, suggesting antioxidant supplementation reduces oxidative stress in the brain (8). Conflicting evidence on the efficacy of antioxidant supplementation highlights the complexities of these compounds in neuroprotection and warrants further research. Specifically, a-LA appears to be sensitive to dose, route, and timing of administration across species (9). While there appear to be benefits to a-LA in brain oxidative stress, it is unclear how the antioxidant and neuroprotective effects of a-LA impact the developing brain.

The brain is one of the most highly metabolic organs in the body. It contains a high concentration of polyunsaturated fatty acids and accumulates redox-active metals, thereby rendering it susceptible to oxidative stress (10). Throughout development, the brain is operating in an increased metabolic state and is rapidly accreting polyunsaturated fatty acids and iron, all while operating with immature antioxidant systems (10). As such, the neonatal brain is likely at an increased risk of experiencing oxidative stress during this dynamic growth period. Because human milk is generally considered to be the optimal source of nutrient provision for the human infant, there exists a need to understand how its components, notably antioxidants, play a role in normal infant development. Analysis of human milk has revealed higher antioxidant capacity compared with infant formulas, possibly suggesting protective effects against oxidative stress exerted through human milk (11, 12). Presently, there appear to be no published reports on a-LA levels in human milk and a limited number of studies investigating the effects of a-LA on neurodevelopment. Provided that a-LA is considered a universal antioxidant, there is interest in understanding whether it may be beneficial in the context of pediatric nutrition.

Use of MRI techniques in the neonatal piglet has previously established the pig as a clinically relevant translatable model for human infants in the context of nutrition and neurodevelopment (13–17). To the best of our knowledge, no study has investigated the effects of a-LA supplementation in infant formula and its effects on the developing brain. Because infants, especially those born preterm, are susceptible to periods of oxidative stress, there exists a need to characterize antioxidants that might be included in infant formulas. As such, the aim of this exploratory study was to examine the dose–response effects of a-LA on growth, cognition, and structural brain development using the preclinical piglet model. We hypothesized that supplementation of this readily absorbed molecule would confer neurodevelopmental changes quantified through magnetic resonance imaging, thus elucidating an optimal dose to support brain development.

## Materials and Methods

All animal care and experimental procedures were in compliance with National Research Council Guide for the Care and Use of Laboratory Animal Care and Use Committee and approved by the University of Illinois Urbana-Champaign Institutional Animal Care and Use Committee.

### Animals and Housing

Thirty-eight vaginally derived intact male Yorkshire piglets from the University of Illinois Imported Swine Research Laboratory were obtained 48 h after birth, to allow for colostrum consumption, and artificially reared over a 27 (replicate 1)- or 29 (replicates 2 and 3)-day trial period. The trial was completed in 3 replicates (12–14 piglets per replicate), with piglets selected from 12 total litters to control for genetics and initial body weight. Piglets were housed individually in stainless steel cages with clear Plexiglass facades and side walls bearing small openings to allow for proper ventilation. Individual piglets had *ad libitum* access to water, as well as a rubber ball and towel for enrichment (16).

Ambient room temperature was maintained between 27 and 29°C and heat lamps provided supplemental heat within the cage. A 12-h light/dark cycle was maintained with light from 0600 to 1800 hours. Prior to placement in the artificial rearing system, piglets were administered 5 ml of *Clostridium perfingens C. perfingens* antitoxin C + D per manufacturer’s recommendations (Colorado Serum company, Denver, CO, USA). Piglets exhibiting sickness behavior, defined as diarrhea for more than 48 h, were placed on an electrolyte solution and administered a single dose of sulfamethoxazole and trimethoprim oral suspension [50 and 8 mg/ml, respectively (Hi-tech Pharmacal, Amityville, NY, USA) for three consecutive days]. Piglets that failed to thrive were euthanized prior to study end.

### Dietary Treatments and Feeding Parameters

All researchers involved with conducting the study and acquiring and analyzing the study results remained blinded to identity of dietary treatments until final statistical analyses had been completed. Piglets were provided either a control (CONT) diet or the control diet supplemented with a low a-LA concentration (LOW) or high a-LA concentration (HIGH) for the duration of the feeding trial. Concentrations of a-LA (Sodium R-Lipoate, GereoNova Research, Inc., Richmond, CA, USA) in test diets were formulated as follows: CONT (0 mg a-LA/100 g milk replacer powder), LOW (120 mg a-LA/100 g milk replacer powder), and HIGH (240 mg a-LA/100 g milk replacer powder). A prebiotic blend of polydextrose (PDX) and galactooligosaccharides (GOS) was provided in all dietary treatments as follows: (1.30 g/100 g milk replacer powder PDX, Danisco, Terre Haute, IN, USA; 2.70 g/100 g milk replacer powder GOS, FrieslandCampina, Zwolle, Netherlands). All dietary treatments were supplemented with docosahexaenoic acid (DHA) (91 mg/100 g milk replacer powder, DSM Nutritional Products, Kingstree, SC, USA) and arachidonic acid (ARA) (182 mg/100 g milk replacer powder, DSM Nutritional Products, Kingstree, SC, USA). Experimental piglet diets were manufactured to human quality standards (Mead Johnson Pediatric Nutrition Institute, Evansville, IN, USA) with adjustments in the nutritional profile made to meet or exceed nutrient requirements for the neonatal pig (18).

All powdered milk replacer formula was reconstituted with 200 g of dry powder per 800 g of water. Given this reconstitution rate, final formulated lipoic acid concentrations (milligram per liter) were as follows: CONT (0 mg a-LA/l), LOW (240 mg a-LA/l), and HIGH (480 mg a-LA/l). On the first day in the artificial rearing system, piglets received small volumes of milk formulas to provide an adjustment period prior to the standard feeding regimen. Piglets were fed at 285, 305, 310 ml of reconstituted diet per kilogram of BW starting on 3, 6, and 12 days of age, respectively. Meal allotment was determined by recording daily body weight of individual piglets. Each diet was reconstituted fresh at each feeding and provided up to five times a day, approximately every 4 h, between 0700 and 2200 hours. Piglets were fasted prior to spatial T-maze assessment to incentivize the milk reward offered in the behavioral task. On days that cognitive assessment occurred (between 17 and 28 days of age) piglets were provided four meals instead of five, while maintaining the aforementioned daily volume per kilogram body weight feeding rate.

### Spatial T-Maze Assessment

Spatial working memory was assessed using a validated behavioral task in a specially designed T-maze for the young pig (19, 20). Beginning at 15 days of age, piglets were subjected to 14 days of behavioral assessment, including an 8-day acquisition phase followed by a 6-day reversal phase. A detailed description of spatial T-maze assessment and piglet performance measures was previously described (15).

### Neuroimaging Procedures

At 29 (replicate 1) and 31 (replicates 2 and 3) days of age, piglets were subjected to magnetic resonance imaging (MRI) procedures. Scanning procedures were implemented on a Siemens MAGNETOM Trio 3 T Imager using a 32-channel head coil. Upon arrival to the Biomedical Imaging Center located in the Beckman Institute for Advanced Science and Technology, piglets were anesthetized *via* intramuscular injection of Telazol (0.07 mg/kg body weight; Zoetis, Florham Park, NJ, USA). Once sedated, piglets were transferred to the MRI scanner and maintained on 2% isoflurane/98% oxygen (Isoflurane, Piramal Healthcare, Bethlehem, PA, USA) for the entirety of the 60-min scan. Piglet vital signs were monitored throughout the scan using an MRI-compatible pulse oximeter, and upon completion of the scan, piglets were maintained under anesthesia and immediately euthanized for tissue collection. Detailed methods for manual brain segmentation, volumetric assessment, voxel-based morphometry, and diffusion tensor imaging were previously described (15, 16).

### Tract-Based Spatial Statistics

The FSL 5.0 toolbox was used for tract-based spatial statistics (TBSS) assessment of fractional anisotropy (FA) data. FA images, previously generated from diffusion data, were manually extracted, and all FA data from individual subjects were aligned using the FSL non-linear registration tool FNIRT. Upon alignment, the study-specific mean FA image was created, and a mean FA skeleton representing the center of all common tracts was established. A threshold of 0.2 was determined to be sensitive for mean FA tract delineation. Once the study-specific mean FA skeleton was created, each subjects’ aligned FA data were projected onto the mean FA skeleton and the resulting voxel-wise cross-subject data were used for statistical analyses (21, 22). For all TBSS analyses involving registration to an atlas, the Piglet Brain Atlas (http://pigmri.illinois.edu/) was used in place of human brain templates (23). For TBSS analysis, only piglets receiving the CONT and HIGH diets were compared. A non-parametric permutation inference function called “randomize” was used within the FSL toolbox and run as a two-sample *t*-test with 500 permutations. Multiple comparisons were also accounted for within the randomize function. The resulting statistical analysis was then presented as heat maps indicating brain areas where FA values differed between dietary treatments.

### Small Intestine Tissue Collection

Immediately following magnetic resonance imaging procedures (described above) and while remaining under anesthesia, piglets were euthanized *via* intracardiac administration of sodium pentobarbital (86.0 mg/kg of body weight; Fatal Plus, Vortech Pharmaceuticals, Dearborn, MI, USA). Following euthanasia, brain and small intestinal tissues were collected. The entire small intestine (i.e., extending from the pyloric sphincter to the ileocecal valve) was removed and immediately measured for total length. The small intestine was further dissected into three segments: duodenum (proximal 10%), jejunum (middle 75%), and ileum (distal 15%). All sections were flushed with saline prior to being weighed, as described previously (24). Data were expressed both on an absolute basis and relative to the final bodyweight of individual piglets.

### Statistical Analysis

An analysis of variance (ANOVA) was conducted using the MIXED procedure of SAS 9.4 (SAS Inst. Inc., Cary, NC, USA) and was applied to differentiate the effects of the CONT, LOW, and HIGH diets provided to young pigs. For outcomes collected at a single time-point (i.e., brain volume and DTI, tissue weight and length), data were analyzed by a one-way ANOVA. Any data collected from the same animal on more than one occasion (i.e., daily bodyweights and behavioral assessment) were analyzed as a two-way, repeated-measures ANOVA. Both statistical models included replicate as a random effects and the level of significance was set at *P* < 0.05 with trends accepted at 0.05 < *P* < 0.10.

## Results

### Piglet Growth and Health

**Figure 1:**
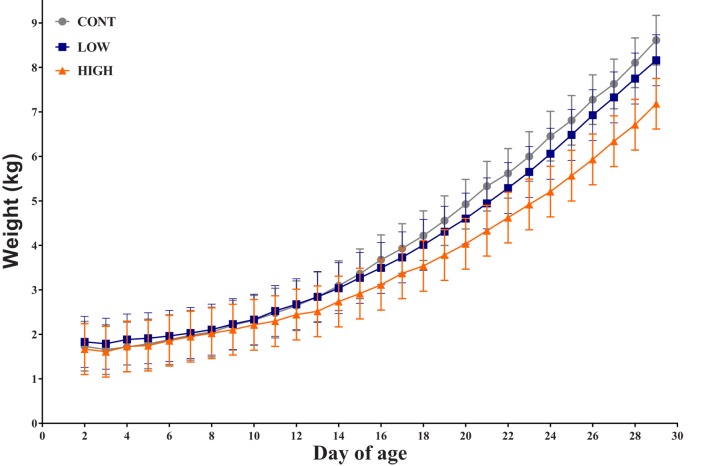
**Piglet daily bodyweight**. Average body weight of each dietary treatment group over 28-day trial period (*n* = 10–11 per treatment); there was no observed interactive effect of treatment × day for daily bodyweight and no main effect of treatment, only a main effect of day was observed (*P* < 0.001).

Overall, there was no observed interactive effect (i.e., diet × day; *P* = 0.229) or the main effect of diet (0.463) for daily bodyweight throughout the feeding trial. However, a main effect of day (*P* < 0.001) was evident (Figure 1). Over the course of this study, seven piglets failed to thrive due to sickness unrelated to dietary treatment (CONT: *n* = 2; LOW: *n* = 2; HIGH: *n* = 3) and as such were euthanized prior to study end. Thus, only 31 piglets are included in the subsequent analyses.

### Intestinal Growth

No main effect of diet was observed for small intestinal length (*P* = 0.949) or total absolute (*P* = 0.783) or relative (*P* = 0.216) intestinal weights (data not shown). Moreover, absolute weight of each small intestinal segment did not differ by diet [duodenum (*P* = 0.711), jejunum (*P* = 0.870), and ileum (*P* = 0.415)], and neither did relative intestinal segment weights [duodenum (*P* = 0.072), jejunum (*P* = 0.154), and ileum (*P* = 0.444)] (data not shown).

### Behavior Performance

Using piglet participation criteria set forth previously (15), four piglets were excluded from behavioral analysis as they displayed at least 3 days of non-compliance during the behavioral testing period. As such, the total number of piglets included in the behavioral analysis was 27 (CONT, *n* = 10; LOW, *n* = 8; HIGH, *n* = 9). Additionally, one piglet (CONT diet) had 1 day of acquisition and 1 day of reversal removed and another piglet (HIGH diet) had 1 day of acquisition removed, all due to non-compliance on those days.

**Figure 2:**
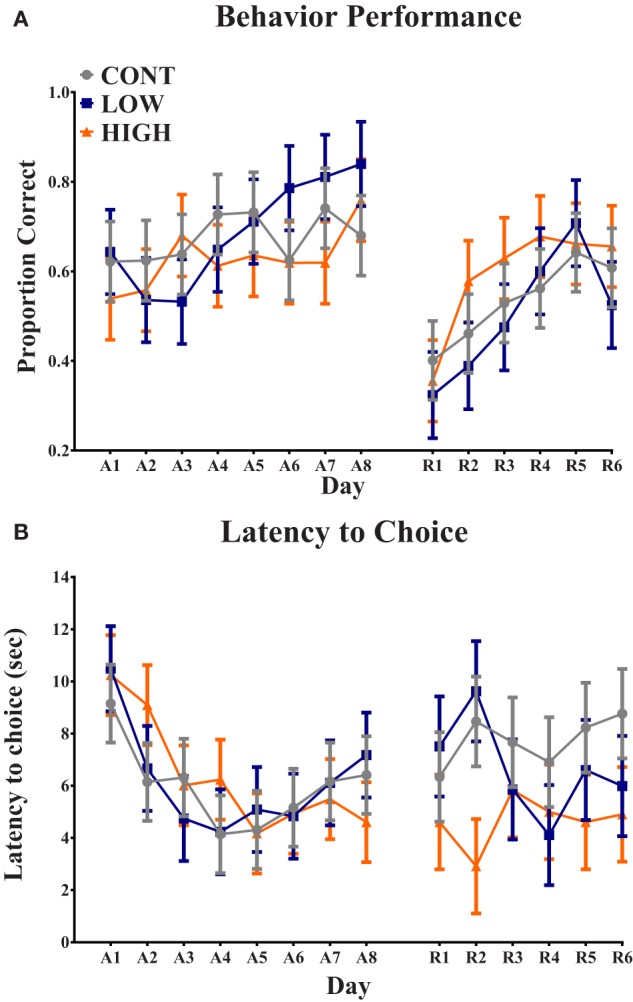
**Spatial T-maze assessment**. **(A)** No diet × day interaction or main effect of diet was observed in proportion of correct choices on each day. Only a main effect of day (*P* < 0.05) was observed for proportion of correct choice. **(B)** No diet × day interaction or main effect of diet was observed in latency to choice on each day. Only a main effect of day (*P* < 0.05) was observed for latency to choice. CONT, LOW, and HIGH treatments were formulated to contain 0, 240, and 480 mg alpha-lipoic acid per liter of reconstituted milk replacer, respectively. (A = acquisition, 8-day phase; R = reversal, 6-day phase).

Analysis of proportion of correct choices during the acquisition phase indicated no interactive effect (i.e., diet × day) (*P* = 0.207) or main effect of diet (*P* = 0.580); only a main effect of day (*P* = 0.019) was observed (Figure 2). Additionally, latency to choice during the acquisition phase exhibited no interactive effect (*P* = 0.720) or main effect of diet (*P* = 0.963); only a main effect of day (*P* = 0.001) was observed. Analysis of proportion of correct choices during the reversal phase also indicated no interactive effect (*P* = 0.637) or main effect of diet (*P* = 0.645); only a main effect of day (*P* = 0.001) was observed. Finally, latency to choice during the reversal phase resulted in no observed interactive effect (*P* = 0.587), main effect of diet (*P* = 0.105), or main effect of day (*P* = 0.824).

### Volumetric Assessment

No differences in absolute total brain volume were observed between dietary treatments (*P* = 0.25; data not shown). Analysis of absolute volumes of individual brain regions also resulted in no volumetric differences due to diet (*P* > 0.247; data not shown). Absolute volumes of individual brain regions were analyzed relative to total brain volume within subject, and relative proportion of total brain volume was analyzed for each region. Dietary treatment tended (*P* = 0.087) to affect cerebellar relative volume, in which piglets provided the HIGH diet had a proportionally larger cerebellum (*P* = 0.037) compared with LOW-fed piglets. No differences due to diet were observed for any relative total volume of individual brain regions (*P* > 0.139).

### Diffusion Tensor Imaging

**Figure 3:**
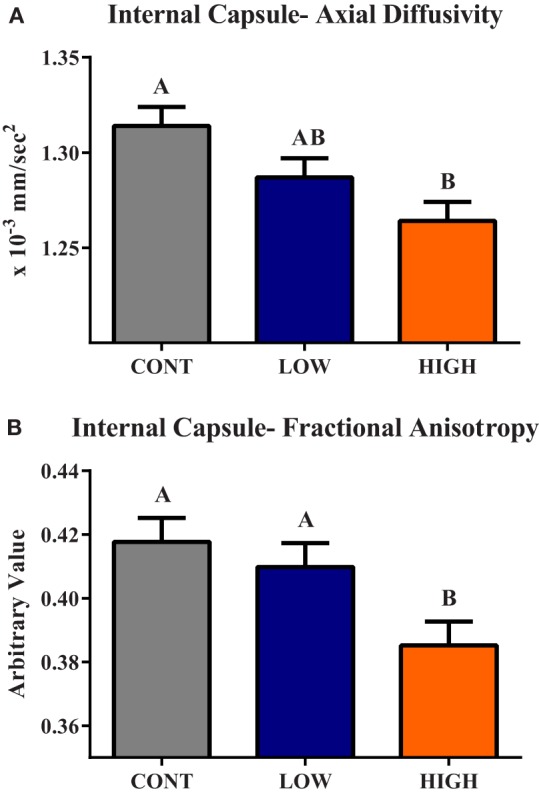
**Internal capsule axial diffusivity and fractional anisotropy**. **(A)** A main effect (*P* < 0.01) of dietary treatment was observed for internal capsule axial diffusivity (AD). **(B)** A main effect (*P* = 0.01) of dietary treatment was observed for internal capsule fractional anisotropy (FA). CONT, LOW, and HIGH treatments were formulated to contain 0, 240, and 480 mg alpha-lipoic acid per liter of reconstituted milk replacer, respectively. ^ab^Means without a common superscript letter differ (*P* < 0.05).

Diffusion tensor imaging revealed a main effect of diet (*P* < 0.05) in the internal capsule axial diffusivity (AD) and FA measures (Figure 3). A main effect (*P* < 0.01) of dietary treatment was observed for internal capsule AD, with piglets provided the HIGH diet exhibiting smaller (*P* < 0.01) AD values compared with CONT piglets, but they were not different (*P* = 0.124) than LOW-fed piglets. Low-fed piglets tended to have smaller (*P* = 0.064) internal capsule AD values when compared with CONT piglets. A main effect of diet (*P* = 0.012) was also observed in internal capsule FA measures. Piglets provided the HIGH diet exhibited lower FA values compared with CONT (*P* < 0.01)- and LOW (*P* = 0.029)-fed piglets. No difference between CONT- and LOW-fed piglets was observed in internal capsule FA values.

The main effect of diet tended to be significant (*P* < 0.10) for AD, radial diffusivity (RD), and mean diffusivity (MD) measures of the thalamus (Table 1). In all cases, no differences between CONT- and LOW-fed piglets were observed. Analysis of thalamic AD values revealed piglets provided the HIGH diet exhibited higher AD values compared with CONT (*P* = 0.031)- and LOW (*P* = 0.054)-fed piglets. Moreover, piglets provided the HIGH diet exhibited higher RD values compared with CONT (*P* = 0.066)- and LOW (*P* = 0.049)-fed piglets. MD values within the thalamus were also noted, with piglets fed the HIGH diet exhibiting higher MD values compared with CONT (*P* = 0.035)- and LOW (*P* = 0.038)-fed piglets. Finally, the main effect of diet tended to be significant for FA values in the cerebellum (*P* = 0.067). Piglets provided the HIGH diet exhibited lower FA values compared with CONT (*P* = 0.036)- and LOW (*P* = 0.052)-fed piglets, but no difference in cerebellar FA values was observed between CONT- and LOW-fed piglets.

**Table 1:** **Thalamic and cerebellar diffusion measures tended to differ (*P* < 0.10) between dietary treatments**.

Region of interest	DTI measure	Diet			Pooled	*P*-value
		CONT	LOW	HIGH	SEM	
Thalamus	AD^c^	1.213^a^	1.218^ab^	1.277^b^	0.021	0.0627
Thalamus	RD^c^	0.750^ab^	0.747^a^	0.783^b^	0.012	0.0904
Thalamus	MD^c^	0.904^a^	0.904^a^	0.948^b^	0.014	0.0569
Cerebellum	FA^d^	0.1851^b^	0.1842^ab^	0.1688^a^	0.0053	0.0666

*CONT, LOW, and HIGH treatments were formulated to contain 0, 240, and 480 mg of alpha-lipoic acid per liter of reconstituted milk replacer, respectively*.

*AD, axial diffusivity; RD, radial diffusivity; MD, mean diffusivity; FA, fractional anisotropy*.

*^ab^Means that do not have a similar letter superscript differ (*P* < 0.05), i.e., “ab” does not differ from “a” or “b”*.

*^c^×10^−3^ mm^2^/s*.

*^d^Arbitrary FA values*.

### Tract-Based Spatial Statistics

**Figure 4:**
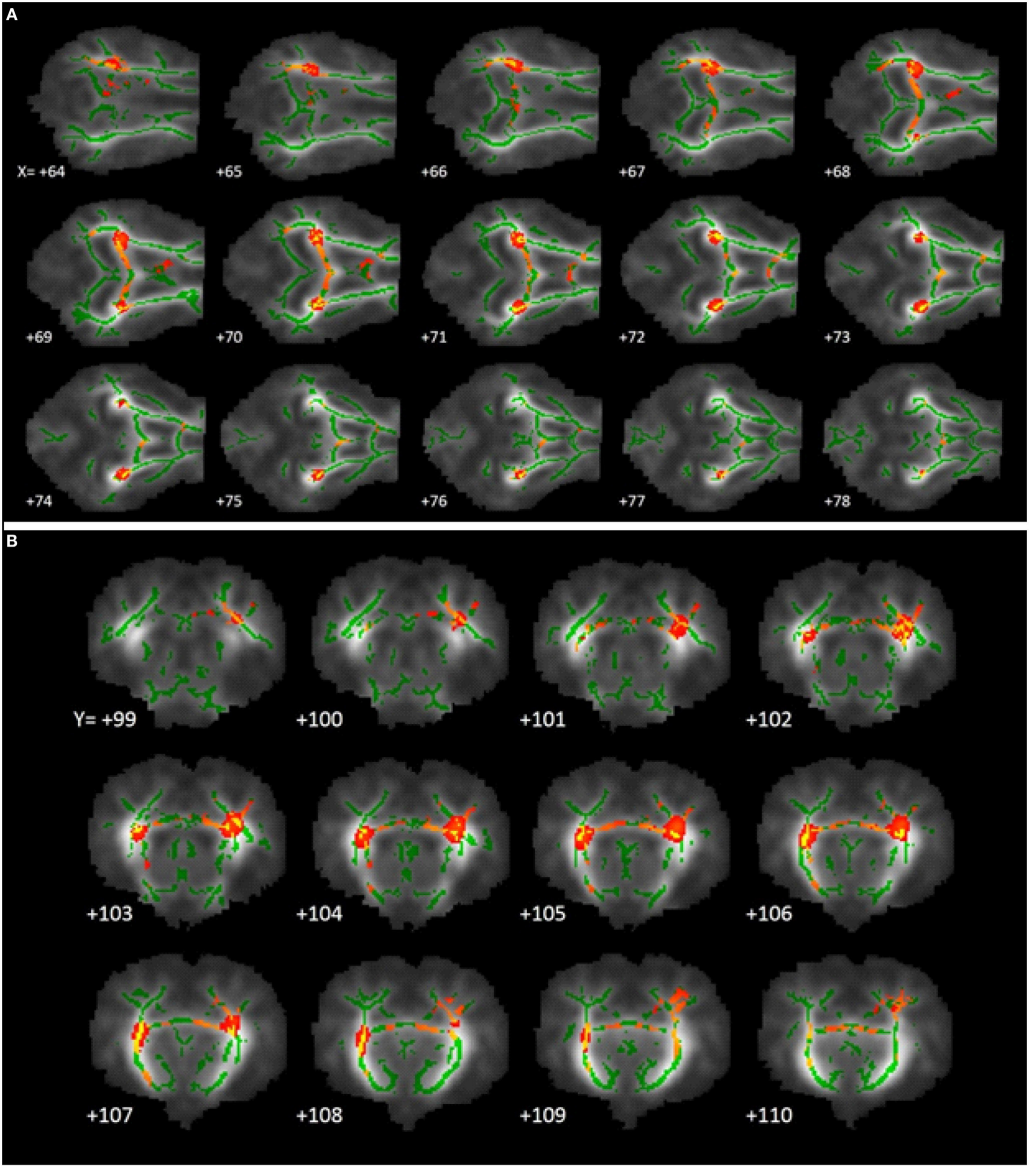
**White matter tract fractional anisotropy differences**. Fractional anisotropy differences along predetermined white matter tracts in which CONT piglets exhibited higher (*P* < 0.05) FA values compared with HIGH piglets. The images generated are an average of all piglet brains from this study, green lines signify regions in which all piglets exhibited white matter voxels. Representative slices were chosen to highlight areas in which FA values in CONT piglets were significantly different when compared with HIGH piglets, noted as red–yellow voxels. **(A)** Axial slices, with varying *X*-coordinates and static *Y* = 103 and *Z* = 110 coordinates, determined using the Piglet Brain Atlas (23). **(B)** Coronal slices, with varying *Y*-coordinates and static *X* = 68 and *Z* = 78 coordinates, determined using the Piglet Brain Atlas. Red and yellow colors indicate degree of statistical difference from *P* = 0.05 to *P* = 0.0001, respectively (23).

Tract-based spatial statistical analysis revealed 1,650 voxels within the predetermined white matter tracts in which CONT piglets exhibited higher (*P* < 0.05) FA values compared with HIGH-fed piglets (Figure 4). There were no significant differences in FA values along the predetermined white matter tracts in which the reverse was true (i.e., where HIGH-fed piglets exhibited higher FA values compared with CONT piglets).

## Discussion

Piglets in this study were supplemented with varying levels of a-LA from 2 to 31 days of age to determine the impact of a-LA supplementation on overall body growth and brain development. Most antioxidant research to date focuses on the effects of supplementation in the context of brain aging, with very little focusing on the needs and efficiency of antioxidants throughout neurodevelopment. It is known that infants, notably preterm infants and those having experienced intrauterine growth restriction, are at an increased risk for oxidative stress due to immature antioxidant systems and elevated metabolic rates, polyunsaturated fatty acid concentrations, and non-protein bound iron concentrations (2, 25). As such, supplementation of antioxidants to alleviate oxidative stress during a highly dynamic growth period might confer benefits to normal growth and development. In our study, we observed that oral supplementation of the HIGH dose of a-LA resulted in altered internal capsule development, suggestive of delayed brain maturation. Moreover, there appeared to be a dose–response with the LOW concentration group being intermediate to the CONT- and HIGH-fed piglets for many outcomes. These data are consistent with other studies of antioxidants in which supplementation of high concentrations appear to exert detrimental effects on neuronal cell cultures (26–28). Although differences in brain development were evident among supplementation groups, provision of a-LA at any level did not affect bodyweight gain or intestinal maturation of piglets. Because there were no gross differences in these measures, we suggest that the observed brain differences were not due to overall piglet size or intestinal maturation of the piglets, rather it was directly due to oral supplementation of a-LA.

In this study, learning and memory were assessed using a spatial T-maze behavioral task. Over the course of the 8-day acquisition period and the 6-day reversal period, there was no observed effect of diet on piglet performance in proportion of correct choices. Moreover, piglet latency to choice also did not differ between dietary treatment groups throughout the acquisition or reversal phases. A main effect of day was observed for proportion of correct choices during acquisition and reversal, indicating learning did in fact occur. These behavioral observations appear to be consistent with previous studies where a-LA supplementation improved memory in aged mice, but not in young mice (6, 7). It should be noted that the young mice used in these studies were 3 months of age, thus being equivalent to an adolescent, rather than neonatal, piglet, or human. However, it was suggested from these studies that supplementation of a-LA may compensate for age-related long-term memory deficits rather than enhancing overall memory in a developing brain.

Diffusion tensor measures revealed differences due to diet in AD and FA within the internal capsule. For both the FA and AD measures, an apparent dose–response was evident with CONT piglets consistently exhibiting the highest values, HIGH piglets exhibiting the lowest values, and LOW piglets being intermediate to the other two treatment groups. Interestingly, analysis of AD and FA in the internal capsule revealed LOW piglets were not different than CONT piglets, suggesting that the LOW dose of a-LA supports normal internal capsule development in the piglet. AD is most commonly described as a measurement of water movement along an axon tract. As development occurs, fiber coherence and myelination increase, thus increasing AD measures. Additionally, FA values tend to increase in brain regions as myelination occurs (29). A recent study in piglets of similar age also suggested alterations in internal capsule development due to dietary intervention, further confirming sensitivity of this brain region to early life nutritional interventions (15). Therefore, the decreased AD and FA values observed in HIGH piglets compared with CONT piglets suggests delayed development within the internal capsule at this time-point.

Thalamic and cerebellar diffusion tensor measures resulted in trending significance that was consistent with observations made in the internal capsule. Cerebellar FA values revealed numerically lower measures in HIGH piglets compared with CONT and LOW piglets. Moreover, RD and MD tend to decrease as white matter matures during development, thus the thalamic RD and MD measures resulted in values indicative of delayed development in HIGH piglets. While these measures are only trending, they are important to highlight because imaging piglets at a later time point may have revealed significant differences between dietary treatments consistent with observations in the internal capsule. Recent work suggests that the internal capsule is the earliest myelinating brain region with other regions starting to myelinate at approximately 6 months of age in the human infant (30). One week of piglet neurodevelopment is equivalent to 1 month of human infant brain development (31, 32). Thus, it would be expected that the internal capsule would be the predominant brain region undergoing maturation at the time imaging occurred in the present study, and imaging at later time-points could reveal greater differences in later-developing regions.

Tract-based spatial statistics comparing CONT- and HIGH-fed piglets revealed differences along white matter tracts that are consistent with diffusion tensor measures described above. Analysis of FA values along the predominant white matter tracts resulted in 1,650 voxels in which CONT piglets exhibited higher measures compared with HIGH-fed piglets. Moreover, visual inspection of these voxels revealed the largest differences occurring in the internal capsule and to a lesser extent the corpus callosum. Together with DTI and TBSS analyses, it is evident that supplementation of high concentrations of a-LA specifically altered development within the internal capsule. Given that the internal capsule is known to be myelinating at this time and these measures indicate HIGH piglets might be experiencing delayed maturation, it is possible that this is due to alterations in myelination status within this region. In the early postnatal period, the brain is rapidly accreting iron due to its pivotal action as a co-factor in myelinating events (33). a-LA is known to chelate transition metals such as iron, and this action is speculated to ameliorate the iron deposition commonly observed in brain aging (5). As such, we speculate that excess of a-LA in the HIGH group may be exerting a negative effect through chelation of accreted iron, resulting in altered myelination status in the internal capsule. Because effects of dietary a-LA on neuroimaging outcomes were observed in pigs receiving the HIGH, but not the LOW, diet, this suggests a threshold may exist whereby oral a-LA supplementation above 240 mg lipoic acid per liter of milk replacer may elicit sufficient iron chelation to alter development of the internal capsule.

To the best of our knowledge, no studies have been conducted to directly assess the effects of oral a-LA supplementation on brain development. Antioxidant research in the context of neurodevelopment appears to be limited to cell culture and suggests that excess supplementation of antioxidants negatively impacts development. Castagné and colleagues proposed that neuronal cells might have an optimal redox status that operates within a tight range of antioxidant capacity (26). They suggest that there is a specific threshold for each antioxidant, below which the compound may confer neuroprotection and above which the compound may become toxic due to an imbalance in redox function toward reduction. Furthermore, this dose–response relationship appears to hold true in chemically unrelated antioxidants, suggesting the neuroprotective effects may be acting through a common mechanism based on redox status of the cell (26). Supplementation of tirilazad mesylate to rat ganglion cells is neuroprotective at low doses but toxic at high doses, and high concentrations of vitamin E succinate are toxic to cultured neurons from the chick embryo retina (27, 28). These data further support the hypothesis that high doses of antioxidants might alter neurodevelopmental processes. As such, our data serve to corroborate the aforementioned *in vitro* findings, further extending the observation of altered brain development due to excess antioxidants in a translational piglet model.

This study is one of the first to assess the impact of a-LA supplementation on the neonatal piglet brain. Supplementation of a-LA at any level had no impact on daily bodyweight, suggesting no detrimental impacts to overall growth or health of the animals. Additionally, no differences in spatial T-maze behavioral assessments were observed, suggesting observed structural differences in the brain may not have functional implications, at least for the cognitive functions required to perform this specific task. Results obtained from DTI and TBSS analyses suggest supplementation of HIGH may have delayed brain development. It is important to note the lack of difference between CONT and LOW piglet neurodevelopmental measures, thereby suggesting a low concentration of a-LA supports normal brain development. Because of the clear dose–response observed in MRI outcomes, future research should be performed at doses closer to the LOW concentration to determine if a-LA supplementation can confer benefits to neurodevelopment. From this study, we conclude that a diet containing a low concentration of a-LA has little impact, whereas a high concentration of a-LA may have delayed structural brain development.

## Author Contributions

RD, RW, and BB were involved in project conceptualization. AM and RD were involved in daily project activities. AM and RD were involved in data collection. AM and RD were involved in data analysis. All authors were involved in data interpretation and manuscript preparation.

## Conflict of Interest Statement

RD received grant funding and has consulted for Mead Johnson Nutrition. RW and BB are employees of Mead Johnson Nutrition.
